# Discrimination in the surgical discipline: an international European evaluation (DISDAIN)

**DOI:** 10.1093/bjsopen/zrab050

**Published:** 2021-06-30

**Authors:** M Holzgang, N Koenemann, H Skinner, J Burke, A Smith, A Young

**Affiliations:** Department of General Surgery, St. James’s University Hospital, Leeds Teaching Hospital Trust, Leeds, UK; UVCM (Visceral Medicine and Surgery), Inselspital Bern, Bern, Switzerland; Department of Trauma Surgery, Orthopaedics, Plastic and Hand Surgery, Augsburg University Hospital, Augsburg, Germany; Department of General Surgery, St. James’s University Hospital, Leeds Teaching Hospital Trust, Leeds, UK; Department of General Surgery, St. James’s University Hospital, Leeds Teaching Hospital Trust, Leeds, UK; Department of General Surgery, St. James’s University Hospital, Leeds Teaching Hospital Trust, Leeds, UK; Department of General Surgery, St. James’s University Hospital, Leeds Teaching Hospital Trust, Leeds, UK

## Abstract

**Background:**

Negative workplace experiences (NWPEs), such as gender discrimination, bullying, sexual harassment and ethnic discrimination, are concerns in today’s surgical society. These negative experiences potentially impair surgeons’ performance and might impact patient care or outcomes negatively. This study aimed to assess the experience of NWPEs across the European surgical workforce.

**Methods:**

A prospective online 34-point questionnaire was designed using a combination of Likert scale, multiple-choice and short-answer questions. Invitations were distributed through surgical associations via email/social media between 1 September and 15 November 2019. Data were analysed using non-parametric methods.

**Results:**

Some 840 complete responses were included in the analysis. The distribution across genders and stage of surgical training was even. Of the respondents, 20 per cent (168 respondents) considered quitting their job, 4.5 per cent (38) took time off and 0.5% (4) left surgery due to NWPEs; 12.9 per cent of females and 4.4 per cent of males experienced some form of physical harassment. Females and those in training were significantly more likely to experience or witness gender discrimination and sexual harassment. Just over half of the respondents (448) did not report negative experiences, with most of these (375 respondents) being unaware of whom to report to. Nearly a fifth of respondents felt that NWPEs influenced patient care or outcomes negatively.

**Conclusion:**

NWPEs were frequent, especially among females and those in training. While a substantial proportion of respondents experienced physical harassment, many individuals were unaware of how to raise concerns. Adverse effects on patient outcomes, surgical training and workforce retention indicate a need for urgent action.

## Teaser

This survey aimed to assess the presence of NWPE (negative workplace experiences summarising gender/ethnic discrimination, bullying and sexual harassment) in the European surgical workforce of today. Its second aim was to assess reporting pathways of NWPE and possible reasons for non-reporting.

## Introduction

Awareness of negative workplace experiences (NWPEs), such as bullying, gender discrimination, ethnic discrimination and sexual harassment, as well as potential adverse effects on surgeons or patients, has risen in the surgical community around the world. Repeated exposure to NWPEs leads to an increase in burn-out, suicidal thoughts and attrition among surgeons^1–3^. Trainees who experience NWPEs are less inclined to consult colleagues or seniors when encountering difficulties, potentially compromising patient care^4^^,^^5^. Bullied trainees are more likely to commit serious or potentially serious medical errors^6^.

Several studies have reported a concerning prevalence of NWPEs in surgery in recent years. Studies from the USA, Australia and the UK have all reported that a significant proportion of surgeons experience discrimination, bullying and undermining behaviours in the workplace^1^^,^^7^^,^^8^, and similar NWPEs have been reported in other specialties^9^^,^^10^.

Despite multiple publications, data from Europe are still relatively scarce. This study aimed to assess the experience and reporting of bullying, gender discrimination, ethnic discrimination and sexual harassment, summarized as negative workplace experiences (NWPEs) across the European surgical workforce.

## Methods

### Survey design

A 34-point online survey (*Fig. S1*) entitled DISDAIN (Discrimination In the Surgical Discipline: An International European evaluatioN) was created with a mix of Likert scale (never = 1, to always = 5), open and short-answer questions, adhering to current survey guidelines^11^^,^^12^. Invitations to participate were distributed by email, social media and newsletters, accompanied by introductory text explaining the purpose of DISDAIN.

All representative surgical societies in Europe as defined by the World Health Organization, (*Fig. S2*) were approached, including all surgical specialties defined by the European Union of Surgical Specialties. Data collection was between 1 September and 15 November 2019 using the SurveyMonkey^®^ platform.

Data sets were excluded if the participant did not practise within Europe or worked outside a defined surgical specialty or if more than three non-demographic questions were not answered.

### Statistical analysis

Data were extracted from SurveyMonkey^®^ (SVMK Inc, California, US) and analysed using Microsoft Excel (MicrosoftCorp- oration^®^Redmond, Washington, US). Median values were calculated and displayed with interquartile ranges. Mann–Whitney calculations were performed to allow comparisons between demographic groups. Statistical significance was defined as *P* < 0.050.

## Results

### Demographics

**Fig. 1:**
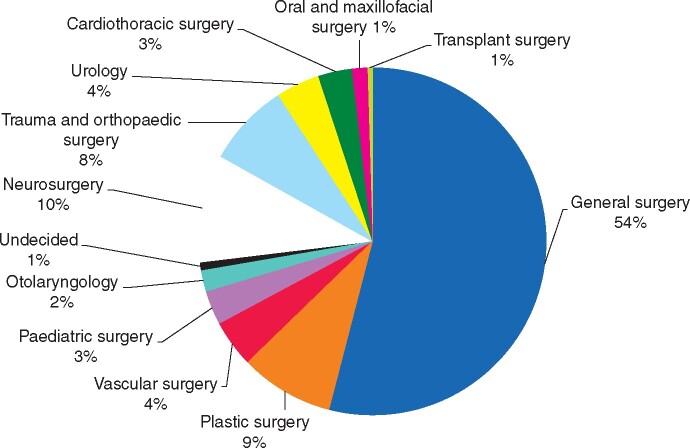
Distribution of surgical specialties

A total of 1038 participants answered the survey. After exclusion of incomplete and non-European responses, 840 data sets were included in the final analysis. The most common reported specialty was general/abdominal surgery (54 per cent), followed by neurosurgery (10 per cent) and plastic surgery (9 per cent; *Fig. 1*). The distribution between genders was even with 50.7 per cent of participants identifying as female and only 13 individuals who preferred not to disclose or did not identify with either gender. The majority of participants identified as Caucasian (81.7 per cent), 10.1 per cent as Hispanic and 2.4 per cent as Middle-Eastern or South Asian. The largest 10-year age group was 31–40-year-olds (41.1 per cent), followed by 41–50-year-olds (26.5 per cent) and 21–30-year-olds (17.9 per cent). Most participants (85.4 per cent) were more than 4 years into their training, with 51 per cent of all participants being independent practitioners or individuals at consultant level.

### Gender discrimination

**Fig. 2:**
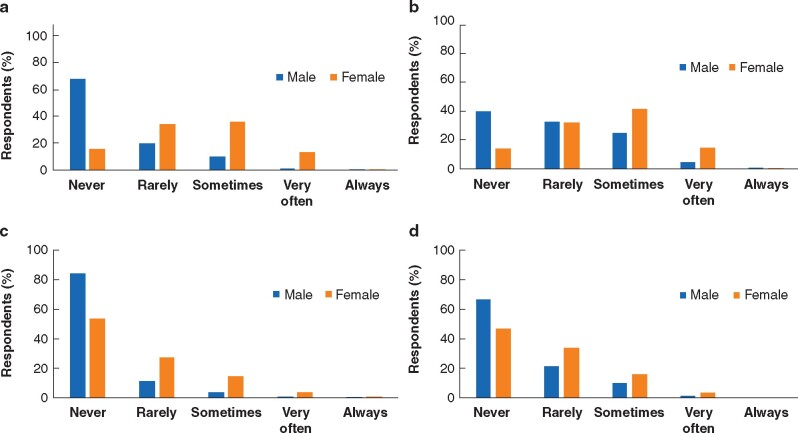
Personally experienced and witnessed gender discrimination and sexual harassment (male *versus* female) **a** Personal experience of gender discrimination. **b** Witnessing of gender discrimination. **c** Personal experience of sexual harassment. **d** Witnessing of sexual harassment

Nearly a third of respondents said they had experienced gender discrimination: 23.3 per cent of respondents described this as ‘sometimes’, 7.5 per cent ‘very often’ and 0.3 per cent ‘always’. Some 41.3 per cent said they had ‘never’ or ‘rarely’ (27.7 per cent) experienced gender discrimination (median Likert score 2.0 (i.q.r. 1.0–3.0)). More than a third of participants stated they had witnessed gender discrimination ‘sometimes’ (32.8 per cent) or ‘very often/always’ (9.3/0.3 per cent), while 25.7 per cent stated this as ‘never’, and 31.6 per cent as ‘rarely’ witnessed (median Likert score 2.0, (i.q.r. 1.5–3.0)). Only 16.3 per cent of females were said to have ‘never experienced’ gender discrimination compared with 68.5 per cent of males. In addition a higher proportion of female respondents (49.0 per cent) also stated having ‘sometimes/very often’ experienced gender discrimination compared with male colleagues (11.4 per cent). Female respondents in this study were significantly more likely to experience personally (median Likert score 2.3 (i.q.r. 2.0–3.0)) as well as to witness gender discrimination (2.7 (i.q.r. 2.0–3.0)) compared with male colleagues (experiencing 1.8 (i.q.r. 1.0–2.0), witnessing 2.0 (i.q.r.1.0–2.7), *P* < 0.001) (*Fig. 2a,b*, *Fig. S3*). Trainee surgeons were more likely to experience and witness gender discrimination (experiencing 2.0 (i.q.r. 1.0–3.0), witnessing 2.3 (i.q.r.1.5–3.0)) compared with senior surgical staff members (experiencing 1.5 (i.q.r. 1.0–2.5) *P* < 0.001, witnessing 2.0 (i.q.r. 1.2–3.0), *P* = 0.003).

### Sexual harassment

Overall, 11.3 per cent of respondents said that they had experienced sexual harassment ‘sometimes/very often/always’ while 88.7 per cent reported’ never/rarely’ having experienced this. Some 15.6 per cent said they had witnessed sexual harassment ‘sometimes/very often/always’. Female surgeons reported both experiencing (median Likert score 1.2 (i.q.r. 1.0–2.0)) and witnessing (1.5 (i.q.r. 1.0–2.0)) sexual harassment more frequently than males (experiencing 1.0 (i.q.r. 1.0–1.0), witnessing 1.0 (i.q.r. 1.0–2.0), *P* < 0.001). While the majority of male colleagues (84.7 and 67.0 per cent respectively) reported never having experienced or witnessed sexual harassment, this applied to less than half (47.0 per cent) of female surgeons. Female independent surgical practitioners and consultants reported significantly higher levels of witnessing sexual harassment than female trainees (median Likert score 2.0 (i.q.r. 1.0–2.0) *versus* 1.3 (i.q.r. 1.0–2.0), *P* = 0.007) (*Fig. 2c,d*).

### Bullying

**Fig. 3:**
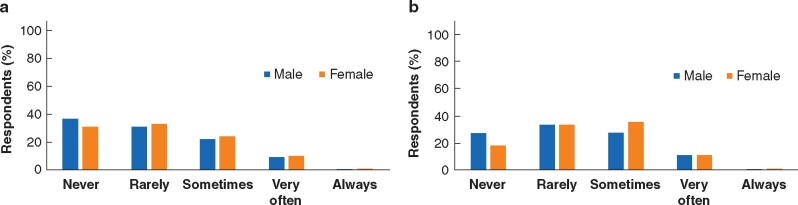
Personally experienced and witnessed bullying (male *versus* female) **a** Personal experience of bullying. **b** Witnessing of bullying

Overall, 23.2 per cent of respondents stated having experienced bullying *‘*sometimes’, 10.3 per cent ‘very often’ and 0.5 per cent ‘always’. Some 43.6 per cent reported they had witnessed bullying (31.5 per cent ‘sometimes’, 11.3 per cent ‘very often’, 0.8 per cent ‘always’) (*Fig. 3a,b*). There was no difference in the personal bullying experience between females and males, although female surgeons were marginally more likely to report witnessing bullying (median Likert score 2.2 (i.q.r. 1.8–3.0)) than their male counterparts (2.0 (i.q.r. 1.3–3.0), *P* = 0.006).

### Ethnic discrimination

Ethnic discrimination (ED) was not widely reported. Eighty-three of 840 respondents said they had sometimes/very often/always experienced ED, 134 of 840 stated to have sometimes/very often/always witnessed ED. There were no differences between Caucasian and non-Caucasian groups nor between males and females, either in experiencing or witnessing this behaviour.

### Role as a surgeon

Overall 85.5 per cent of respondents stated that they felt taken seriously by patients in their role as a surgeon *‘*very often/always’. Females, however, felt taken significantly less seriously (median Likert score 4.0 (i.q.r. 4.0–5.0)), than their male counterparts (5.0 (i.q.r. 4.0–5.0), *P* < 0.001). While 78.7 per cent of all participants felt that were taken seriously ‘very often/always’ by male colleagues and 84.7 per cent by female colleagues, female surgeons said they were taken significantly less seriously by both male (4.0 (i.q.r. 3.0–4.0) *P* < 0.001), and female colleagues (4.0 (i.q.r. 4.0–5.0) *P* = 0.009), compared with the male respondents (5.0 (i.q.r. 4.0–5.0)).

Females also reported that they were more likely to be addressed in inappropriate terms by male colleagues (median Likert score 2.0 (i.q.r. 1.5–3.0)) than male respondents (1.0 (i.q.r. 1.0–2.0), *P* < 0.001). Females were significantly more likely to refrain from engaging in tasks/learning opportunities due to sexist comments (2.0 (i.q.r. 1.0–2.0)) compared with males (1.0 (i.q.r. 1.0–1.0), *P* < 0.001).

### Types of NWPEs

Some 12.9 per cent of females and 4.4 per cent of males reported some form of physical NWPE. The majority (57.5 per cent females, 48.6 per cent males) reported verbal statements alone as the most common form of NWPEs (*Fig. S3*).

### Reporting incidents

**Fig. 4:**
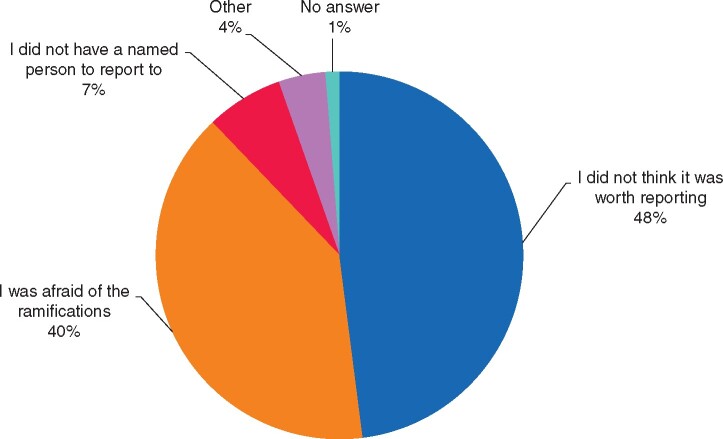
Reasons for not reporting negative workplace experiences

Nearly half of the respondents stated they had no knowledge of a designated person in their institution to report NWPEs to, while a quarter stated they were unsure. Only 12.2 per cent of respondents stated having reported an incident after experiencing/witnessing it. When asked for reasons for not reporting, the most common answers were ‘feeling incident was not worth reporting’, ‘being afraid of the ramifications/consequences’ and ‘not having a designated person to report to’ (*Fig. 4*).

### Potential influence of NWPEs

Nearly a fifth of all respondents (18.8 per cent) stated that they felt their NWPEs that they had experienced or witnessed had a negative effect on patient care, safety or outcome. Some 20 per cent of respondents (168 respondents) said that such negative experiences had made them consider quitting surgery and 38 individuals (4.5 per cent) reported having taken time off work due to NWPEs. Of these, four stated that NWPEs were the reason for leaving surgery.

## Discussion

This study aimed to assess the experience of NWPEs in the surgical workforce across Europe. The results raise a genuine concern. There should be zero tolerance of the behaviours outlined (bullying, sexual harassment, gender or ethnic discrimination), yet one in five participants considered quitting surgical training or practice due to NWPEs. The disparity between those considering leaving the workforce (168) and those who had done so^5^, suggests that many might be enduring repeated NWPEs, leading to dissatisfaction and potentially negative effects on performance. It is also likely that surgeons who have already left the workforce would not have been reached by this survey, so the proportion leaving the specialty may have been higher than the figure obtained.

Nearly a fifth of participants in this survey were concerned about the potential negative effects of NWPEs on patient care, safety and outcomes. There are few data on this subject, although a study from 2015 showed that a surgical trainee who has experienced NWPEs was less likely to report concerns or seek help in regards to patient care, increasing potential risk for patient safety^5^. A recent observational study including more than 13 000 patients showed that patients of surgeons who were more frequently reported for NWPEs by their colleagues were significantly more likely to experience postoperative complications^13^.

Physical NWPEs were experienced by 12.9 per cent of female and 4.4 per cent of male respondents in this survey. A study in 2019 surveyed 173 plastic surgery trainees and found that 19.9 per cent reported to have experienced sexual harassment and 3.6 per cent reported being physically abused during their training. In around two thirds of cases, the source of sexual harassment was identified as the attending physician^14^. Although other studies have found lower rates of physical abuse (2.2 per cent) with similar frequencies in men and women, attending surgeons were still cited as the most common perpetrators^1^. The percentage of physical abuse cited in the present survey was higher than these earlier reports^1^^,^^14^. In addition there is the likelihood that physical mistreatment may be under-reported.

The present study found female surgeons to be significantly more likely to experience or witness gender discrimination or sexual harassment than males. Females also felt they were taken significantly less seriously, were addressed inappropriately more frequently by their colleagues, and were more likely to refrain from learning opportunities due to sexist comments. Conversely, no male respondents felt that sexist comments made them refrain from learning opportunities. Other studies investigating gender differences across varying surgical specialties have also found that female surgeons reported higher incidences of sexual harassment and discrimination^15–17^.

Senior female surgeons reported significantly higher levels of witnessing sexual harassment than female trainees. This might reflect professional experience or that they become sensitized to the subject over time. Gender discrimination on the other hand, was experienced and witnessed more by trainees than senior surgeons. These findings have also been reported elsewhere^16^^,^^18^.

The survey did not show clear evidence of discrimination on the basis of ethnicity, although this may reflect the fact that over 80 per cent of respondents were Caucasian. It was not possible to determine whether non-Caucasians were identified or responded across Europe with the same frequency as the Caucasian majority.

Concerning reporting pathways, many participants stated that they were unaware or unsure of a designated person or institution to report to. Only a minority of surgeons who experienced or witnessed an adverse event reported it; reasons for failure to do so include ‘not thinking it is worth reporting’ or ‘fear of ramifications’. Other studies have reported similar findings^17^^,^^19^. Available data suggest that affected individuals feel either afraid of consequences or that reporting NWPEs is an unnecessarily cumbersome exercise.

This study has limitations. Access to information and contacts in the different countries varied. The identification of surgical society websites in the different European nations was difficult as many were incomplete or inaccessible. The survey was sent out to the different societies in each nation where every society was asked to distribute it to their membership. The extent to which organizations complied and distributed the survey is unknown and, consequently, so is the total number of individuals who might have responded. Variation in internet presence among different surgical societies might also explain the unequal percentages of participants from different countries. This heterogeneity meant that statistical comparisons between countries and regions were not made. As the survey was conducted only in English, some potential responders may have been discouraged from participating and incomplete responses may have existed due to language barriers.

Selection bias exists at a number of levels. Distribution of the survey via social media was biased towards people who are more active in digital medical networks. As colleagues were encouraged to distribute the survey in their departments, it is possible that individuals with higher personal motivation in regard to the topic were recruited. Considering the efforts to address the survey to the European surgical community as a whole, the actual return rate was relatively low, reflecting a further risk for selection bias. Due to the voluntary nature of participation, it is likely that individuals with strong personal feelings or experiences in regard to NWPEs will be over-represented. SurveyMonkey^®^ was used as the data collection tool due to its user-friendliness. This would allow respondents potentially to answer the questionnaire more than once, although this was not considered particularly likely.

All of these limitations probably contribute to the paucity of multinational literature looking at NWPEs, as opposed to national studies^8^^,^^14–16^^,^^19^^,^^20^. Despite this, the present study has shown that NWPEs are commonplace throughout the European surgical workforce and should not be dismissed or underestimated. NWPEs remain under-reported, mainly as a result of insufficient reporting pathways or fear of retribution. Affected individuals need to be provided with safe, trustworthy and efficient reporting pathways, ideally outside their own surgical department. Only an increase in awareness of NWPEs through reporting can lead to a long-term cultural change and result in zero-tolerance policies for harmful behaviours.

*Disclosure*. The authors declare no conflicts of interest.

## Supplementary material

Supplementary material is available at *BJS Open* online

## Supplementary Material

zrab050_Supplementary_Data

## References

[1] Hu Y-Y , Ellis RJ, Hewitt DB, Yang AD, Cheung EO, Moskowitz JT et al Discrimination, abuse, harassment and burnout in surgical residency training. N Engl J Med 2019;381:1741–1752 doi:10.1056/NEJMsa1903759 31657887 10.1056/NEJMsa1903759 PMC6907686

[2] Zhang LM , Ellis RJ, Ma M, Cheung EO, Hoyt DB, Bilimoria KY et al Prevalence, types and sources of bullying reported by US General Surgery Residents in 2019. JAMA 2020;323:2093–2095 doi:10.1001/jama.2020.2901 32453357 10.1001/jama.2020.2901 PMC7251443

[3] Wall M , Schenck-Gustafsson K, Minucci D, Sendén MG, Løvseth LT, Fridner A et al Suicidal ideation among surgeons in Italy and Sweden – a cross-sectional study. BMC Psychol 2014;2:53 25520811 10.1186/s40359-014-0053-0 PMC4266411

[4] Huang Y , Chua TC, Saw RPM, Young CJ. Discrimination, bullying and harassment in surgery: a systematic review and meta-analysis. World J Surg 2018;42:3867–3873 doi:10.1007/s00268-018-4716-5 29971462 10.1007/s00268-018-4716-5

[5] Wild JRL , Ferguson HJM, McDermott FD, Hornby ST, Gokani VJ. Undermining and bullying in surgical training: a review and recommendations by the Association of Surgeons in Training. Int J Surg 2015;23:S5–9 doi:10.1016/j.ijsu.2015.09.017 26369864 10.1016/j.ijsu.2015.09.017

[6] Paice E , Smith D. Bullying of trainee doctors is a patient safety issue. Clin Teach 2009;6:13–17. doi:10.1111/j.1743-498x.2008.00251

[7] Crebbin W , Campbell G, Hillis DA, Watters DA. Prevalence of bullying, discrimination and sexual harassment in surgery in Australasia. ANZ J Surg 2015;85:905–909 26510837 10.1111/ans.13363

[8] Clements JM , King M, Nicholas R, Burdell O, Elsey E, Bucknall V et al Bullying and undermining behaviors in surgery: a qualitative study of surgical trainee experience in the United Kingdom (UK) and Republic of Ireland (ROI). Int J Surg 2020;84:219–225.doi:10.1016/j.ijsu.2020.07.031 32738542 10.1016/j.ijsu.2020.07.031

[9] Pololi LH , Brennan RT, Civian JT, Shea S, Brennan-Wydra E, Evans AT. Us, Too. Sexual harassment within academic medicine in the United States. Am J Med 2020;133:245–248 31301297 10.1016/j.amjmed.2019.06.031

[10] Jenner S , Djermester P, Prügl J, Kurmeyer C, Oertelt-Prigione S. Prevalence of sexual harassment in academic medicine. JAMA Intern Med 2019;179:108–111 30285070 10.1001/jamainternmed.2018.4859 PMC6583418

[11] Jones TL , Baxter MA, Khanduja V. A quick guide to survey research. Ann R Coll Surg Engl 2013;95:5–7 23317709 10.1308/003588413X13511609956372 PMC3964639

[12] Eysenbach G. Improving the quality of Web surveys: the Checklist for Reporting Results of Internet E-Surveys (CHERRIES). J Med Internet Res 2004;6:e34 15471760 10.2196/jmir.6.3.e34 PMC1550605

[13] Cooper WO , Spain DA, Guillamondegui O, Kelz RR, Domenico HJ, Hopkins J et al Association of coworker reports about unprofessional behavior by surgeons with surgical complications in their patients. JAMA Surg 2019;154:828–834 31215973 10.1001/jamasurg.2019.1738 PMC6585020

[14] D’Agostino JP , Vakharia KT, Bawa S, Sljivic S, Natoli N. Intimidation and sexual harassment during plastic surgery training in the United States. Plast Reconstr Surg Glob Oben 2019;7:e2493 10.1097/GOX.0000000000002493 PMC6964922 32042539

[15] Capek L , Edwards DE, Mackinnon SE. Plastic surgeons: a gender comparison. Plast Reconstr Surg 1997;99:289–299 9030134 10.1097/00006534-199702000-00001

[16] Dresler CM. Experiences of women in cardiothoracic surgery. A gender comparison. Arch Surg 1996;131:1128–1134 8911251 10.1001/archsurg.1996.01430230010002

[17] Smeds MR , Aulivola B. Gender disparity and sexual harassment in vascular surgical practice. J Vasc Surg 2020;**72**:692–699 32067879 10.1016/j.jvs.2019.10.071

[18] Ling M , Young CJ, Shepherd HL, Mak C, Saw RPM. Workplace bullying in surgery. World J Surg 2016;40:2560–2566 27624759 10.1007/s00268-016-3642-7

[19] Nayyar A , Scarlet S, Strassle PD, Olila DW, Erdahl LM, McGuire KP et al *A National Survey of Sexual Harassment Among Surgeons - Abstract. Academic Surgical Congress Abstracts Archive*. https://www.asc-abstracts.org/abs2019/85-06-a-national-survey-of-sexual-harassment-among-surgeons/. (accessed September 2019).

[20] Halim UA , Riding DM. Systematic review of the prevalence, impact and mitigating strategies for bullying, undermining behavior and harassment in the surgical workplace. Br J Surg 2018;105:1390–1397 30007001 10.1002/bjs.10926

[21] United Nations, Department of Economic and Social Affairs. *Countries of Europe, Population Division, World Population Prospects: The 2017 Revision*. https://www.un.org/en/development/desa/population/migration/data/index.asp (accessed May 2019).

[22] World Health Organization. *Countries of Europe.* http://www.euro.who.int/en/countries (accessed May 2019)

[23] European Union of Medical Specialists. Officially Registered Surgical Specialties. https://www.uems.eu/about-us/medical-specialties (accessed May 2019).

