# Supplementary material for: Discrimination in the surgical discipline: an international European evaluation (DISDAIN)
Source: BJS Open. 2021 Jun 30;5(3):zrab050. doi: 10.1093/bjsopen/zrab050 (PMC8242223; doi:10.1093/bjsopen/zrab050)
Supplement: zrab050_Supplementary_Data [file zrab050_supplementary_data.docx]

**Supporting information, Figure S1 (Questionnaire)**

**DISDAIN QUESTIONNAIRE**

1. Which country do you currently work in?

(Dropdown list of all countries of Europe)

1. To which gender identity do you most identify?

- Male

- Female

- Prefer not to say

1. What is your age group?

- 21-30

- 31-40

- 41-50

- 51-60

- Above 60

1. What ethnicity do you mostly identify with?

- Caucasian

- Hispanic

- Middle-Easter

- East-Asian

- South-Asian

- Mixed

- Black

- Other

5. What is your current stage of training

- Below 2 years

- 2-4 years after graduation

- 4-6 years after graduation

- Over 6 years after graduation

- Consultant/Independent Surgeon

6. What is your chosen Surgical Specialty

- Cardiothoracic

- General Surgery

- Neurosurgery

- Oral and Maxillofacial Surgery

- Otolaryngology

- Pediatric Surgery

- Plastic/Aesthetic Surgery

- Trauma and Orthopedic Surgery

- Transplant

- Urology

- Vascular

- Haven’t decided yet

7. Which specialties have you previously worked in?

- Cardiothoracic

- GeneralAbdominal Surgery

- Neurosurgery

- Oral and Maxillofacial Surgery

- Otolaryngology

- Pediatric Surgery

- Plastic/Aesthetic Surgery

- Trauma/Orthopedic Surgery

- Transplant Surgery

- Urology

- Vascular Surgery

- Other

8. In my current of previous specialties I personally experienced gender discrimination:

- Never (Likert 1)

- Rarely

- Sometimes

- Very Often

- Always (Likert 5)

9. In my current or previous specialties I witnessed gender discrimination amongst

colleagues:

- Never (Likert 1)

- Rarely

- Sometimes

- Very Often

- Always (Likert 5)

10. In my current or previous specialties I personally experienced bullying:

- Never (Likert 1)

- Rarely

- Sometimes

- Very Often

- Always (Likert 5)

11. In my current or previous specialties I witnessed bullying amongst colleagues:

- Never (Likert 1)

- Rarely

- Sometimes

- Very Often

- Always (Likert 5)

12. In my current or previous specialties I personally experienced sexual harassment:

- Never (Likert 1)

- Rarely

- Sometimes

- Very Often

- Always (Likert 5)

13. In my current or previous specialties I witnessed sexual harassment amongst colleagues:

- Never (Likert 1)

- Rarely

- Sometimes

- Very Often

- Always (Likert 5)

14. In my current or previous specialties I personally experienced ethnic discrimination:

- Never (Likert 1))

- Rarely

- Sometimes

- Very Often

- Always (Likert 5)

15. In my current or previous specialties I witnessed ethnic discrimination amongst

colleagues:

- Never (Likert 1)

- Rarely

- Sometimes

- Very Often

- Always (Likert 5)

16. In my current or previous specialties I personally experienced discrimination for being

pregnant or maternity/paternity leave:

- Never (Likert 1)

- Rarely

- Sometimes

- Very Often

- Always (Likert 5)

17. In my current or previous specialties I witnessed discrimination for being pregnant or for taking maternity/paternity leave:

- Never (Likert 1)

- Rarely

- Sometimes

- Very Often

- Always (Likert 5)

18. If you have experienced Sexual Harassment/Bullying/Discrimination can you specify  which type?

- Verbal

- Written

- Social Media

- Phyiscal

- Other

19. If you have experienced Sexual Harassment/Bullying/Discrimination have you ever reported it?

- Yes

- No

- Not applicable

20. In your opinion was the matter taken seriously by respective authorities?

- Yes

- No

21. Why did you not report it?

- I did not have a named person to report to

- I was afraid of the consequences/ramifications

- I did not think it was worth reporting

- Other

22. Is there a designated person to talk to about issues of discrimination/bullying/sexual harassment at your hospital?

- Yes

- No

- Unsure

23. Have you ever experienced or witnessed bullying/sexual harassment or discrimination

and felt that it had affected patient care/safety or outcome?

- Yes

- No

24. Have you ever taken time off work because of discrimination/bullying/sexual

harassment?

- Yes

- No

25. Have you ever considered quitting surgical training because of discrimination/bullying/

sexual harassment?

- Yes

- No

- This was the reason I quit

26. Have you ever considered changing your surgical specialty because of discrimination, bullying or sexual harassment?

- Yes

- No

- I have changed because of this

27. In general do you feel taken seriously by your patients in your role as a surgeon?

- Never (Likert 1)

- Rarely

- Sometimes

- Very Often

- Always (Likert 5)

28. In general, do you feel taken seriously by your male colleagues in your role as a  surgeon?

- Never (Likert 1)

- Rarely

- Sometimes

- Very Often

- Always (Likert 5)

29. In general, do you feel taken seriously by your senior female colleagues in your role  as a surgeon?

- Never (Likert 1)

- Rarely

- Sometimes

- Very Often

- Always (Likert 5)

30. Have you been addressed by inappropriate terms by male colleagues?

- Never (Likert 1)

- Rarely

- Sometimes

- Very Often

- Always (Likert 5)

31. Have you been addressed by inappropriate terms by female colleagues?

- Never (Likert 1)

- Rarely

- Sometimes

- Very Often

- Always (Likert 5)

32. Are you ever assigned stereotypically male tasks at work?

- Never (Likert 1)

- Rarely

- Sometimes

- Very Often

- Always (Likert 5)

33. Are you ever assigned stereotypically female tasks at work?

- Never (Likert 1)

- Rarely

- Sometimes

- Very Often

- Always (Likert 5)

34. Have sexual/discriminating comments ever made you refrain from engaging in work tasks/learning opportunities?

- Never (Likert 1)

- Rarely

- Sometimes

- Very Often

- Always (Likert 5)

35. Comments/Experiences

**Supporting information, Figure S2**

**Definitions of terms (alphabetical order)**

*Bullying (B*): seeking to harm, intimidate or coerce, emotionally or physically (https://definitions.uslegal.com)

*Ethnic discrimination (ED):* a situation in which someone is treated less well because of their ethnicity (https://definitions.uslegal.com)

*Gender Discrimination (GD*): a situation in which someone is treated less well because of their gender (<https://dictionary.cambridge.org/dictionary/english/gender-discrimination>)

Europe: the definition of “Europe” is not uniform. According to the perspective through which one talks about the continent (geographical, political, economical etc) different countries will be in- or excluded. For the purpose of this study we predominantly adhered to a list of 44 European countries as per statistics of the United Nations (17). These countries include Russia, Germany, United Kingdom, France, Italy, Spain, Ukraine, Poland, Romania, Netherlands, Belgium, Czech Republic, Greece, Portugal, Sweden, Hungary, Belarus, Austria, Serbia, Switzerland, Bulgaria, Denmark, Finland, Slovakia, Norway, Ireland, Croatia, Moldova, Bosnia and Herzegowina, Albania, Lithuania, North Macedonia, Slovenia, Latvia, Estonia, Montenegro, Luxembourg, Malta, Iceland, Andorra, Monaco, Liechtenstein, San Marino and Holy See. As no surgical societies could be identified in Andorra, San Marino and Holy See these countries dropped out of the evaluation. However, Georgia, Armenia, Azerbaijan, Turkey, and Cyprus were added as European countries according to the WHO (18). As such 46 countries were left to be contacted.

*Sexual harassment (SH):* the making of unwelcome and inappropriate sexual remarks or physical advances (in a workplace) (<https://dictionary.cambridge.org/dictionary/english/sexual-harassment)>

Surgical specialties: the surgical specialties questioned in our survey were defined according to the European Union of Medical Specialists (19). The following eleven surgical specialties were therefore included in the survey: cardiothoracic surgery, general surgery, neurosurgery, oral and maxillofacial surgery otolaryngology, pediatric surgery, Plastic/Aesthetic/Reconstructive Surgery (including hand surgery), Transplant Surgery, Trauma and Orthopedic Surgery, Urology and Vascular Surgery.

**Supporting information Figure S3 (free comments, selected samples)**

“Bullying usually comes following a hierarchical pattern: you are bullied by your superiors and you tend to bully your subordinates (even though most of times you don't even realize you have "bully attitudes")”

“A trainer also wrote to my dean stating that women were not physically strong enough to be orthopaedic surgeons.”

“Discrimination at such high level of intellect and skills is very subtle and difficult to substantiate or demonstrate.”

“All different surgery specialties have very specific requirements and needed abilities where one or other gender would have advantage. Women have better dexterity and ability to work with small things, so naturally there is a higher number of women in ophthalmic surgery field. But when You have to operate 150kg patient in abdominal or orthopedic field, you need raw physical strength that most women can not provide.”

“I have to say that there are also discrimination not only from male colleages, but also from patients, escpecially elderly male patients, they assume that because I am a female that I am a nurse not a doctor.”

“I was told as a Core Surgical Trainee that if I wanted to be taken seriously as a surgeon I should stop wearing dresses to work. I’ve been told to ‘snuggle up’ when scrubbed assisting a senior male colleague in theatre as surgery is a ‘contact sport’.”

“In my department, that is mostly male, I think females are usually treated better than same level male colleagues, mostly in terms of better surgical trainings opportunities and an easier access to congresses and scientific meetings.”

“Ladies are given many privileges and extra comforts in surgery, and generally easier time, which I don't have issues with. However sometimes the positive discrimination is obvious and favoritism shown.”
